# The “Beam-Me-In Strategy” – remote haptic therapist-patient interaction with two exoskeletons for stroke therapy

**DOI:** 10.1186/s12984-019-0547-3

**Published:** 2019-07-12

**Authors:** Kilian Baur, Nina Rohrbach, Joachim Hermsdörfer, Robert Riener, Verena Klamroth-Marganska

**Affiliations:** 1 Sensory-Motor Systems Lab, Department of Health Sciences and Technology, Swiss Federal Institute of Technology (ETH Zurich), Tannenstrasse 1, Zurich, 8092 Switzerland; 2 Spinal Cord Injury Center, University Hospital Balgrist, University of Zurich, Zurich, Switzerland, Forchstrasse 340, Zurich, 8008 Switzerland; 3 Chair of Human Movement Science, Department of Sport and Health Sciences, Technical University of Munich, (TU Munich), Munich, Germany, Georg-Brauchle-Ring 60-62/III, Munich, 80992 Germany; 4 School of Health Professions, Institute of Occupational Therapy, Zurich University of Applied Sciences (ZHAW), Winterthur, Switzerland, Technikumstrasse 81, Winterthur, 8400 Switzerland

**Keywords:** Telerehabilitation, Teleassessment, Haptic interaction, Robot-assisted rehabilitation, Robot-assisted assessment, Neurorehabilitation, Stroke, Stroke rehabilitation, Bidirectional teleoperation, Exoskeleton, Arm rehabilitation

## Abstract

**Background:**

We present a robot-assisted telerehabilitation system that allows for haptic interaction between therapist and patient over distance. It consists of two arm therapy robots. Attached to one robot the therapists can feel on their own arm the limitations of the patient’s arm which is attached to the other robot. Due to the exoskeleton structure of the robot, movements can be performed in the three-dimensional space.

**Methods:**

Fifteen physical and occupational therapists tested this strategy, named “Beam-Me-In”, while using an exoskeleton robot connected to a second exoskeleton robot in the same room used by the study experimenter. Furthermore, the therapists assessed the level of impairment of recorded and simulated arm movements. They quantified four typical impairments of stroke patients: reduced range of motion (active and passive), resistance to passive movement, a lack of ability to fractionate a movement, and disturbed quality of movement.

**Results:**

On a Likert Scale (0 to 5 points) therapists rated the “Beam-Me-In” strategy as a very useful medium (mode: 4 points) to evaluate a patient’s progress over time. The passive range of motion of the elbow joint was assessed with a mean absolute error of 4.9^∘^ (absolute precision error: 6.4^∘^). The active range of motion of the elbow was assessed with a mean absolute error of 4.9^∘^ (absolute precision error: 6.5^∘^). The resistance to passive movement (i.e. modified Tardieu Scale) and the lack of ability to fractionate a movement (i.e. quantification of pathological muscle synergies) was assessed with an inter-rater reliability of 0.930 and 0.948, respectively.

**Conclusions:**

The “Beam-Me-In” strategy is a promising approach to complement robot-assisted movement training. It can serve as a platform to assess and identify abnormal movement patterns in patients. This is the first application of remote three-dimensional haptic assessmen t applied to telerehabilitation. Furthermore, the “Beam-Me-In” strategy has a potential to overcome barriers for therapists regarding robot-assisted telerehabilitation.

## Introduction

Typical upper limb impairments after stroke are muscle weakness with reduced range of motion (ROM), spasticity, reduced ability to fractionate movements, reduced movement smoothness and deviation from an intended movement path [1–3]. Physical and occupational therapists provide long-term senorimotor rehabilitation training to reduce functional impairment.

Rehabilitation robots support and enhance physical or occupational therapy. They can deliver therapy with high intensity and provide quantitative assessments [4–8]. Additionally, robotic devices can assess abnormal movement patterns related to the impairment of an individual [9–12]. The devices enhance motivation through games and tasks that are performed on a graphical display. More and more clinics implement rehab gyms where several devices are provided and allow several individuals to train in one room. This setting enables individuals also to train in multiplayer settings, meaning that the devices are connected and individuals train together by playing one game, either with each other or against each other [13]. Multiplayer games provide diversified game play and incorporate social interaction to promote enjoyment of the involved players. The role of the therapist during robot-assisted training and robot-assisted assessment is often restricted to set parameters and supervise the training that the device provides. Notwithstanding, a physical or occupational therapist is indispensable for neurorehabilitation therapy as the therapist determines the course of the treatment and surveys the course of recovery. Through interview, clinical observation, and movement guidance (i.e., manually moving the arm of the patient), the therapist gathers relevant information and interprets it in order to establish limitations, refine the diagnosis and guide the therapy [14].

Both, therapist and patient, interact with the rehabilitation robot during robot-assisted therapy. A robotic device that is accepted by the therapist will indirectly satisfy the patient. In surveys on therapist acceptance of technical devices for therapy, 91% of the therapists quoted the desire for hands-on therapy as a barrier for the use of technologies [15]. 96% rated the option to get feedback from a device as important or very important. Most therapists agreed that biofeedback on muscle activation (71%) and joint position (54%) would be a useful tool for them [16]. In robot-assisted therapy, the therapist can visually estimate joint positions but only gather limited information regarding muscle activation. The lack of movement guidance by the therapist in robot-assisted therapy makes a haptic identification of the muscle activation impossible. Furthermore, the quality of clinical observation communicated by the robotic system to the therapist is limited. The potential of robotic-systems in assessments of the patient to improve sensitivity and provide biofeedback is already identified [17]. In current implementations, the biofeedback is provided as quantified information assessed by the robot (e.g., numbers on screen). These numbers may not facilitate refinement of diagnosis and guidance of therapy. To our knowledge, no literature comparing different modalities of feedback (visually, auditory, haptically) regarding their impact on refinement of diagnosis does not exist. We suggest to facilitate the use of robots by therapists by providing the biofeedback in an intuitive way (e.g., haptically). The added value of robot-assisted therapy for the therapist could be a novel experience of the patient’s capabilities.

To provide such a biofeedback, the device must capture independent, concurrent and precise information on the position and torques in each single segment of the arm (i.e., the upper arm, the lower arm and the hand). As each exoskeleton segment is attached to the corresponding arm segment of the patient, haptic feedback, i.e. one desired element of biofeedback, can be transmitted by exoskeleton robots. And this information could be transmitted from one device to the other not only to allow multiplayer gaming of two indidivudals for training but also to allow interaction of the therapist with an individual.

We present an application where an exoskeleton robot enables the therapists to feel the patient’s limitations in their own arm and, thus, provides a completely new way of patient-therapist interaction. We call it the “Beam-Me-In” strategy. We implemented it in ARMin, an exoskeleton robot that was developed for senorimotor neurorehabilitation of the arm [4, 18, 19]. The ARMin robot assesses and haptically presents kinetic and kinematic functions of each single joint (i.e., shoulder, elbow, and wrist) in the three-dimensional space. “Beam-Me-In” is realized through use of two ARMin robots. Kinematic functions are assessed by the position sensors on one robot and are presented on the second robot (i.e., a unidirectional design of a master-slave system [20]). The kinetic reaction in the second, guided robot can be assessed by force sensors and fed back to the first robot as an interaction force. We present a bidirectional master-slave system between two devices (i.e., two ARMins) with 7 degrees of freedom each, that provides haptic reification of the patient’s impairments (ARMin 1) to the therapist’s arm (ARMin 2) and thus, provides technology that enables the therapist to be “beamed” into the patient [21].

Our study aimed to test if patient behavior can be transmitted over exoskeleton rehabilitation robots to provide a “Beam-Me-In” experience to therapists. In order to evaluate how far therapists can experience the patient’s disability, we determined how accurately, reliably, and confidently therapists can quantify patient’s motor impairments by having their arm actively or passively moved through the patient’s trajectory and then estimating outcomes based on the therapist’s own proprioception and vision.

## Methods

**Fig. 1:**
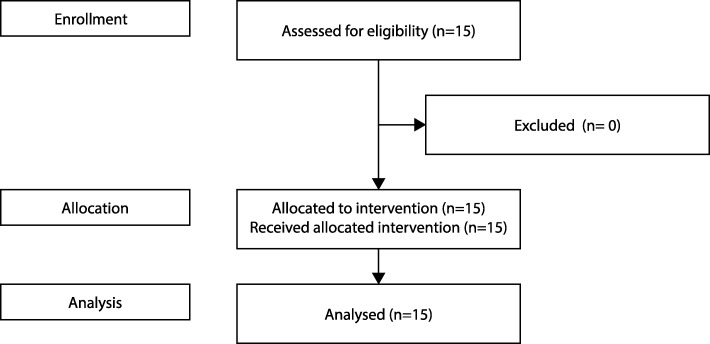
The study flow chart

This clinical study with 15 participants took place at the Sensory Motor Systems Lab at ETH Zurich, Switzerland, from July to August 2015 (Fig. 1).

### ARMin

**Fig. 2:**
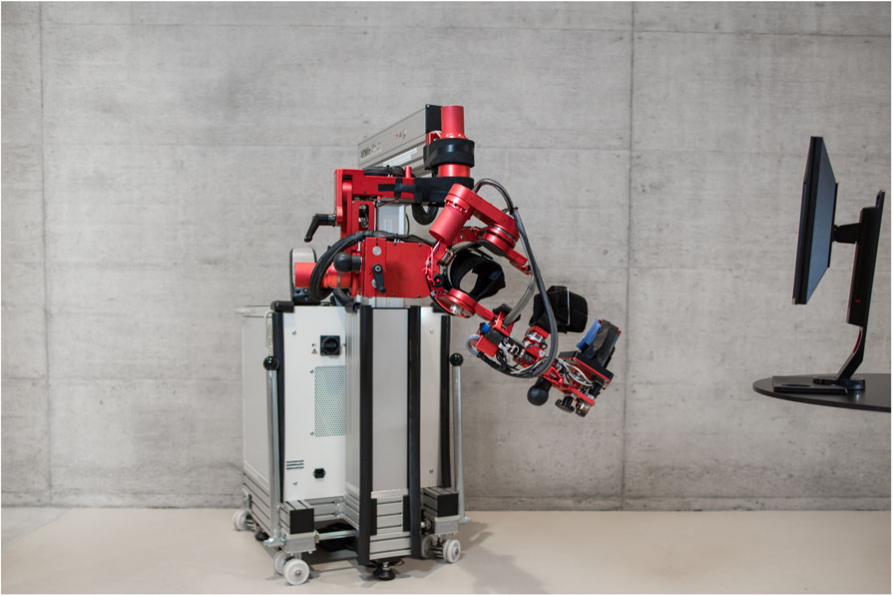
ARMin arm rehabilitation robot (Generation IV)

In the exoskeleton robot ARMin, the three main mechanical segments of the patient arm (i.e., upper arm, lower arm and hand) are attached with cuffs to the three corresponding segments on the ARMin robot. Each cuff is equipped with a 6-degrees-of-freedom (DOF) force sensor measuring the interaction forces between patient and robot. The connection of the three segments to the robot basis represents seven DOF of the human arm: 3D shoulder rotation, elbow flexion/extension, pro/supination of the lower arm, wrist flexion/extension and hand opening and closing. The joints are actuated and their rotational angle is measured by potentiometers and encoders (Fig. 2).

 The ARMin robot can be adjusted to the patient by changing the length of the segments and the height of the robot according to the patient’s characteristics. The robot can be easily switched from a right to a left side configuration. Mechanical end stops are providing patient safety. In order to minimize interaction forces felt by the patient (i.e., maximize transparency of the robot), design and control of the robot were optimized. The robot is designed for negligible backlash, backdrivable motors and is compensated in gravity and friction [22]. Therefore, the robot is highly transparent. The forces measured at the force sensors are reduced to the patient-robot interaction forces[18].

### Participants

Therapists were recruited by personal contact among clinics collaborating with ETH Zurich. Therapists were included if they were aged 18 years or older and were certified physical or occupational therapists with a minimum of three years of basic education. Therapists were excluded 1) if their own passive range of motion was less than 120^∘^/0^∘^/0^∘^ for elbow flexion/extension and less than 140^∘^/0^∘^/0^∘^ for shoulder elevation according to the neutral zero method or 2) if they had a neurological, orthopedic, rheumatologic, or other disease restricting movements of the tested arm or 3) if they had a pacemaker or other implanted electronic devices. All participants had to sign an informed consent. The responsible ethical committee approved the study (KEK-ZH-Nr. 2015-0013, Zurich, Switzerland).

### Course of action

One experimenter conducted the practical part of the clinical study. At start of the session, each participant answered questions regarding professional background and opinion regarding 1) the relevance of technical devices in rehabilitation, 2) the relevance of the human component in therapy and rehabilitation, 3) the usage of robots in physical/ occupational therapy and 4) the importance of interaction between therapist and robots in therapy (for questions, see Results, Table 2).

For the assessment, the participant’s arm was attached to ARMin. First, the participant received an introductory training by the experimenter. It started with four minutes of passive mobilization (=participant-passive) in one ARMin device. Next, the bidirectional master-slave system with two ARMins was tested by the participant with the experimenter as second actor. The bidirectional master-slave system was tested during three minutes in the master mode (=participant-active), and three minutes in the slave mode (=participant-passive).

**Fig. 3:**
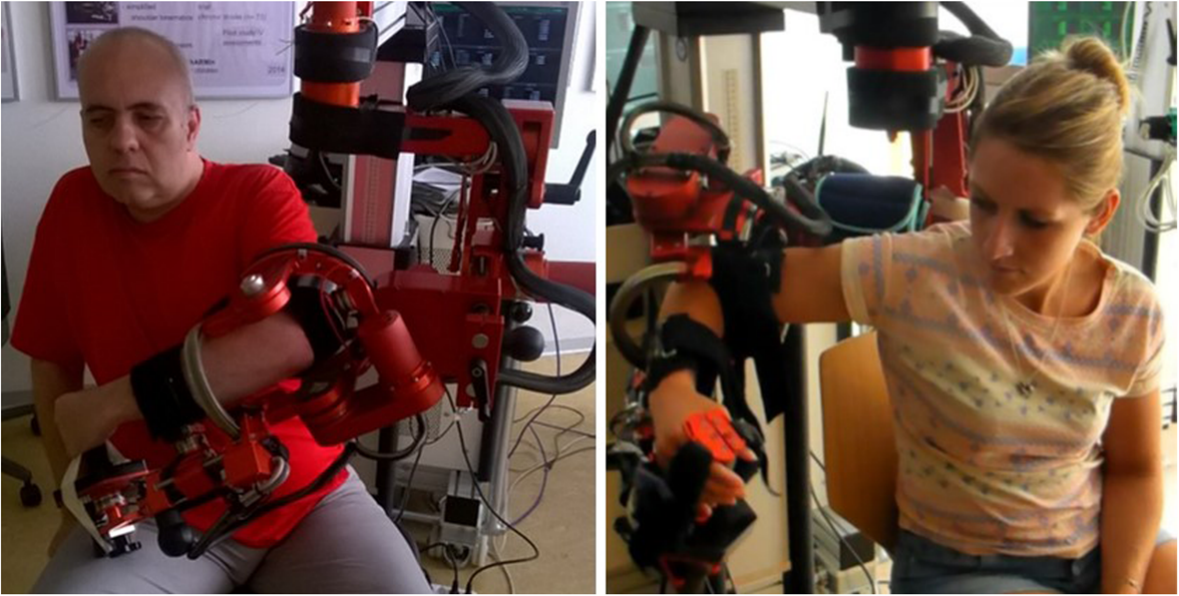
Recording and replaying the QOM assessment. (Left) Stroke patient recorded while performing the QOM task in transparent mode. (Right) Participant in slave mode, experiencing the replayed QOM performance (mirrored to the participant’s dominant side)

Four tasks for examination of a paretic arm were chosen to allow for assessment after stroke: active and passive ROM, resistance to passive movement (RPM), pathological muscle synergies (SYN) and quality of movement (QOM). For data acquisition for each of these four tasks, either recordings from a real subject were used, or subjects were simulated and then replayed during the study (Fig. 3). This ensured standardized conditions for each participant. The ARMin robot provides encoder resolutions below 0.005^∘^ which facilitates a high repeatability of simulated joint movements. Simulated end effector positions are repeatable within a range of 0.5 mm [19].

The four tasks ROM, RPM, SYN and QOM were presented in the same order and the subjects’ movement of each task was presented in randomized order to each participant. The participant drew from envelopes that contained the different randomized sequences of subjects’ movement. The envelopes were prepared by the experimenter. The participant was not aware of the differences between the sequences. The participant evaluated the performance of subjects for each of the four tasks by quantification of the subject’s performance using clinical scores, and answered task-related questions. After the assessment of the four tasks with ARMin, the participant filled in a closing questionnaire, which rated overall impression and fields of application of the “Beam-Me-In” strategy.

### Clinical tests

#### ROM

##### Data acquisition

Three subjects with different active (aROM) and passive ROM (pROM) in elbow joint were simulated (1. aROM 15^∘^-110^∘^, pROM 0^∘^-120^∘^; 2. aROM 50^∘^-90^∘^, pROM 20^∘^-110^∘^; 3. aROM 40^∘^-85^∘^, pROM 30^∘^-105^∘^).

##### Procedure

To introduce the task, the participant was passive while the elbow joint was flexed and extended in ARMin by the experimenter in intervals of 5 degrees from 0^∘^ to 120^∘^ and the participant was verbally informed about each 5^∘^-step and could look at the arm position. Afterwards, the participant could freely move through the ROM for one minute to explore the limits. The participant was allowed to feel each of three simulated subjects (aROM: participant passive; pROM: participant active) ten times, and then quantified the aROM and pROM with a required 5^∘^ resolution. The three different ranges for aROM and pROM were used to differentiate severity among the different subjects.

#### RPM

##### Data acquisition

To evaluate muscle tone, the resistance to passive movement during passively induced flexion/extension was simulated in ARMin for three different subjects. Three subjects with varying degrees of impairment according to the “modified Tardieu Scale” (mTS) in the arm were simulated. The mTS is a clinically established test which assesses the muscle’s response to stretch at given velocities in degrees per second, and the quality of muscle reaction on an ordinal scale ranging from 0 to 4 (with “0” meaning “no spasticity”) [23]. Subject 1 represented a healthy person (mTS=0, pROM 0^∘^ to 120^∘^, no speed threshold, no catch angle, no stiffness, no damping). Subject 2 represented a mildly affected person with a slight resistance of the elbow flexor muscles which was simulated by an increase in damping as soon as a certain speed threshold in extension was exceeded (mTS: 1, pROM: 20^∘^ to 110^∘^, speed threshold: 80^∘^/s, no catch angle, no stiffness, damping: 1 Nms/^∘^). Subject 3 represented a severely affected person post-stroke where the movement was interrupted at a certain angle (“catch angle”) when a predefined speed threshold was reached (mTS: 2, pROM: 30^∘^ to 105^∘^, speed threshold: 40^∘^/s, catch angle: 60^∘^, stiffness: 0,3Nm/^∘^, no damping).

##### Procedure

The participant was allowed to feel each of the three simulated subjects ten times. First, the participant quantified pROM (participant active) with a required 5^∘^ resolution. Then, the angle of muscle reaction, if present, was quantified and the quality of muscle reaction was rated following the common instructions of the mTS [24]. The assessment of the three different levels of resistance to passive movement was used to differentiate severity among the different subjects. Since the same three pROMs as in the ROM task were assessed and range of motion is part of mTS, the results of ROM and RPM were compared to test for intra-rater reliability.

#### SYN

##### Data acquisition

**Fig. 4:**
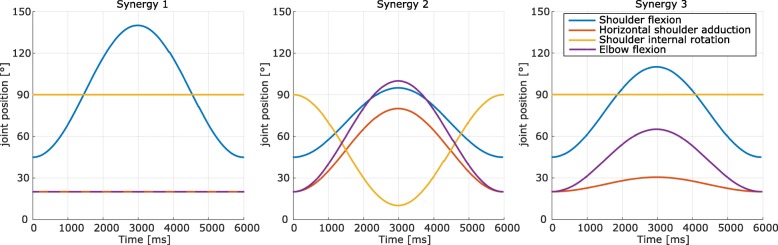
Simulated movement of subjects 1 (left), 2 (middle) and 3 (right) for SYN task. Subject 1: 110^∘^ of pure shoulder flexion, no additional elbow movement. Subject 2 and 3: Reduced shoulder flexion with additional shoulder abduction/external rotation and elbow flexion

An upper extremity flexor synergy can typically be observed in voluntary flexory arm movements [25]. Components of a flexor synergy were experimentally quantified in previous studies [26–28]. While healthy subjects are able to selectively move one joint while keeping the other segments still (interjoint coordination), patients post stroke commonly lose this capability and present a flexion synergy pattern with abduction and external rotation of the shoulder together with flexion of elbow, hand and fingers [29]. To assess the ability of the participant to distinguish between a normal, selective movement and a loss of the inter-joint coordination resulting in a pathological muscle synergy, arm movements of three simulated subjects were presented to the participant. They were created based on movement profiles of a healthy subject (subject 1), and subjects post-stroke (subjects 2 and 3). For all three movements, the same starting position and a sinusoidal-type position-controlled movement with a period of 6 s duration was chosen (Fig. 4).

##### Procedure

The participant behaved passively. First, all three arm movements were haptically presented to allow for comparison by the participant. Afterwards, each movement was presented three times and had to be rated for “selectivity” (i.e., ability to fractionate the movement) on a 6-point Likert scale (0=“not selective at all” to 5=“normally selective”) [30]. The assessment of the three different simulated arm movements was used to differentiate severity among the different subjects.

#### QOM

##### Data acquisition

Path accuracy and smoothness were used as indicators for quality of movement. To record the data, subjects were instructed to move a cursor (end-effector of ARMin) as directly and smoothly as possible in a 2-DOF point-to-point reaching task on the graphic display. Path accuracy was calculated as the distance to path ratio [4]. A value of one represents a straight line; higher values imply a less accurate path. Movement smoothness was calculated as the arc length of the movement speed profiles’ normalized Fourier magnitude spectrum [31]. A smoothness value close to -2.8 was considered as “optimal”, lower values implied less smooth movement. An optimal trajectory was simulated and used as standard. Three trajectories of healthy subjects and a trajectory of a post-stroke subject with severe disability were recorded and haptically presented to the participant using the robot (Table 1). The strong variance in duration of the healthy subjects’ movements is to be considered.

**Table 1:** Subjects for QOM assessment

Subject	Smoothness [-]	D-P ratio [-]	Duration [s]
Stroke (recorded)	-4.40	1.28	9.0
Healthy A (recorded)	-3.15	1.00	10.5
Healthy B (recorded)	-3.61	1.02	5.0
Healthy C (recorded)	-3.92	1.03	8.5
Perfect (simulated)	-2.80	1.00	10.0

**Table 2:** Characteristics of the participants (N=15)

mean age in years:	30.4 (SD 8.0)
gender female, male: N	14, 1
mean professional experience in years:	5.1 (SD 5.3)
professional experience with therapy robots:	
None: N	1
little (<10 h): N	5
moderate (10-50 h): N	7
high (>50 h): N	2
professional experience with ARMin or Armeo Power:	
None: N	10
little (<10 h): N	4
moderate (10-50 h): N	1
high (>50 h): N	0
How relevant do you judge technical devices in rehabilitation? Mode (SD)*	4 (0.70)
How relevant do you judge the human component in therapy and rehabilitation? Mode (SD)*	5 (0.51)
How convinced are you about the importance of robots in physical/ occupational therapy? Mode (SD)*	4 (0.74)
Do you think the interaction between therapist and robots in therapy is of importance? Mode (SD)*	4 (0.59)

*0 = not at all; 1 = not very; 2 = mildly; 3 = moderately; 4 = very; 5 = extremely

**Table 3:** Summary of the ROM differentiation based on the assessment given by the participants (N=15)

	aROM			AVG
Presented span of joint motion to be differentiated	95^∘^ >40^∘^ (*Δ*=55^∘^)	95^∘^ >45^∘^ (*Δ*=50^∘^)	45^∘^ >40^∘^ (*Δ*=5^∘^)	
Correct differentiation	15/15 = 100.0%	15/15 = 100.0%	10/15 = 66.7%	88.9%
	pROM			
Presented span of joint motion to be differentiated	120^∘^ >90^∘^ (*Δ*=30^∘^)	120^∘^ >75^∘^ (*Δ*=45^∘^)	90^∘^ >75^∘^ (*Δ*=15^∘^)	
Correct differentiation	14/15 = 93.3%	15/15 = 100.0%	15/15 = 100.0%	97.8%

(AVG=average, aROM/pROM=active/passive range of motion)

##### Procedure

The participant was passive. First, the optimal trajectory was presented five times with visual feedback on the screen. Then, the subjects’ movements were presented haptically in randomized order, separated by a “washout”, presenting the optimal trajectory without visual feedback. The participant rated smoothness and movement accuracy on a 6-point Likert scale (0=“not at all” to 5=“normally smooth/accurate”). The assessment of the four different arm movements was used to differentiate severity among the different subjects.

#### Task related questions

The statement “I am confident about my assessment results” was rated on a 6-point Likert scale (0=“strongly disagree” to 5=“fully agree”) regarding aROM, pROM and RPM. The statements “I experienced the patient‘s capabilities at my own arm” and “I felt beamed in the patient” were rated on a 6-point Likert scale (as above, 0 to 5) for all four tasks (i.e., ROM, RPM, SYN, QOM).

#### End-of-study questions

At the end, i.e., after the assessment of the four tasks in ARMin, the participant answered questions regarding the session and his/her own opinion about the “Beam-Me-In” strategy and its applicability in telerehabilitation therapy (for the questions, see Results, Table 16).

#### Statistical analysis

The feature extraction for the QOM task and the data analysis were performed using MATLAB (Mathworks, R2014b). For all four tasks, the number of correct patient rankings regarding severity was assessed. The performance in quantifying the ROM angles was analyzed by mean absolute errors and mean precision errors (i.e., the standard deviation of a set of measurements) [32]. The mTS scoring in (RPM), the SYN scoring, and the QOM scoring were analyzed regarding intra-class correlation coefficient (ICC). The ICC was used to establish the inter-rater reliability for the values indicated by the participants. A two-way mixed model analysis with absolute agreement was performed to test the consistency of the scores. The ICC values were interpreted according to Cicchetti (0.00 -0.39 (poor), 0.40 - 0.59 (fair), 0.60 - 0.74 (good) and 0.75 - 1.00 (excellent) [33].

The one sample Wilcoxon signed-rank test (*α*=.05) was used to estimate the difference of the ROM medians, to determine whether these differed from the presented data, and to compute the intra-rater reliability of pROM indicated by the therapists in ROM and in RPM. The Wilcoxon test tested the null hypothesis that the average signed rank of the two dependent samples (ROM and RPM) was zero.

For the task related questions, mean, mode and standard deviation were assessed. The answers in the task related questions were correlated to the performances of the raters and coefficient of determination and p-values were calculated.

For the end-of-study questions, mean, mode and standard deviation were assessed.

## Results

### Participants

Fifteen adults participated in the study (for characteristics, see Table 2).

### Clinical tests

#### ROM

The number of correctly differentiated angles in ROM averaged 93.3% (Table 3). The mean absolute error in identifying each single angle averaged 4.9^∘^ with an absolute precision error of 6.5^∘^ (Table 4).

**Table 4:** Summary of the ROM quantification given by the participants (N=15)

	aROM						
	Extension			Flexion			AVG
Presented angle [^∘^]	15	50	40	110	90	85	
Mean absolute error [^∘^]	4.7	8.3	7.0	4.0	2.3	3.3	4.9
Absolute precision error [^∘^]	6.5	8.3	8.4	5.6	4.0	5.6	6.4
	pROM						
Presented angle [^∘^]	0	20	30	120	110	105	
Mean absolute error [^∘^]	2.0	5.7	6.3	2.0	5.3	8.0	4.9
Absolute precision error [^∘^]	3.7	7.3	7.1	6.5	7.0	7.4	6.5

(AVG=average, aROM/pROM=active/passive range of motion)

#### RPM

**Fig. 5:**
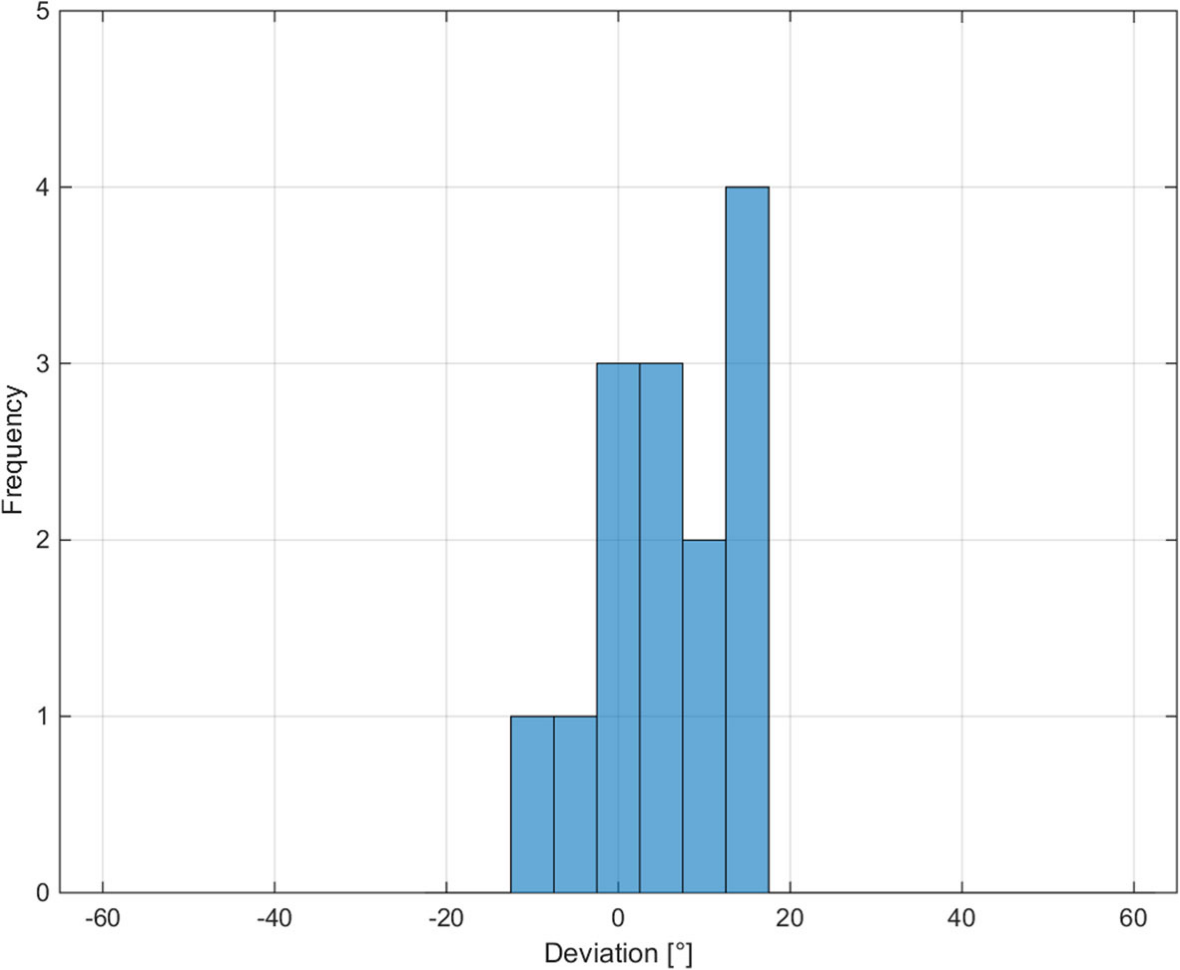
Deviation distribution of the stated catch angles compared to the simulated catch angle of 60^∘^ elbow extension. (N=14, mean absolute error=11.3^∘^, mean precision error=16.0^∘^)

The number of correctly scored mTS averaged 93.3% (Table 5). One participant did not identify the catch of the simulated severely affected subject 3 and was excluded for the evaluation of the catch angle quantification (Fig. 5 and Table 6). The two way mixed effects model showed excellent intra-class correlation (according to Cicchetti (1994), Table 7).

**Table 5:** Results of the RPM quantification

mTS Scoring	Subject 1 mTS=0	Subject 2 mTS=1	Subject 3 mTS=2
0	14 (participants)	0	0
1	1	14	1
2	0	1	14
3	0	0	0
4	0	0	0
Mode	0	1	2

Three simulated subjects with different modified Tardieu Scale (mTS) (0 “healthy” to 2 “severely affected”) were scored by participants (N=15) on the mTS (0 to 4)

**Table 6:** Results of the catch identification (N=14)

Catch identification	14/15 participants
Mean absolute error (absolute precision error), N=14	7.9^∘^ (8.1^∘^)

**Table 7:** Intra-class correlation of “mTS-Scores” between participants (N=15)

	Intra-Class Correlation	95% CI	F Test with True Value 0	
			Value	Sig
Single measures	0.930	0.757 to 0.998	201	0.000
Average measures	0.995	0.979 to 1.000	201	0.000

Two-way mixed effects model where both people effects and measures effects are random, CI=Confidence Interval

#### SYN

All 15 participants could distinguish the severely affected, mildly affected and healthy subjects (all simulated). The quantification of the performances regarding severity illustrates the participants’ skill to distinguish between different movement synergies (Table 8). The intra-class correlation was excellent (according to Cicchetti (1994), Table 9).

**Table 8:** Results of the SYN quantification given by the participants (N=15)

	Simulated subject		
Response	Healthy	Mildly affected	Severely affected
Not at all (0)	0 (participants)	0	7
Rarely (1)	0	0	8
Somewhat (2)	0	3	0
Moderately (3)	0	11	0
Fairly normal (4)	8	1	0
Normally (5)	7	0	4
Mode	4	3	1

**Table 9:** Intra-class correlation of “Synergy-Scores” between participants (N=15)

	Intra-Class Correlation	95% CI	F Test with True Value 0	
			Value	Sig
Single measures	0.948	0.811 to 0.998	275	0.000
Average measures	0.996	0.985 to 1.00	275	0.000

Two-way mixed effects model where both people effects and measures effects are random, CI=Confidence Interval

#### QOM

The number of correctly differentiated QOM performances averaged 73.3% for smoothness and 91.1% for accuracy (Table 10). The participants quantified the subjects’ smoothness and accuracy (Table 11). The intra-class correlation was fair (according to Cicchetti (1994), Tables 12 and 13).

**Table 10:** Results of the QOM differentiation

Presented performances to be differentiated	Patient < Healthy A	Patient < Healthy B	Patient < Healthy C	AVG
Smoothness: Correct differentiations	14	12	7	73.3%
Accuracy: Correct differentiations	14	14	13	91.1%

(N=15)

**Table 11:** Results of the QOM quantification (N=15). Four subjects (three healthy and one patient) were rated regarding smoothness and accuracy

	Simulated Subject							
	Patient		Healthy A		Healthy B		Healthy C	
Response	Smoothness	Accuracy	Smoothness	Accuracy	Smoothness	Accuracy	Smoothness	Accuracy
Not at all (0)	2	6	0	0	2	0	3	0
Not very (1)	9	5	0	0	1	3	5	5
Mildly (2)	4	4	3	4	1	2	4	4
Moderately (3)	0	0	6	2	5	4	2	5
Very (4)	0	0	5	9	5	6	1	1
Extremely (5)	0	0	1	0	1	0	0	0
Mode	1	0	3	4	3	4	1	1

**Table 12:** Intra-class correlation of “Quality of Movement-Scores Smoothness” between participants (N=15)

	Intra-Class Correlation	95% CI	F Test with True Value 0	
			Value	Sig
Single measures	0.475	0.177 to 0.931	14.59	0.000
Average measures	0.931	0.764 to 0.995	14.59	0.000

Two-way mixed effects model where both people effects and measures effects are random, CI=Confidence Interval

**Table 13:** Intra-class correlation of “Quality of Movement-Scores Accuracy” between participants (N=15)

	Intra-Class Correlation	95% CI	F Test with True Value 0	
			Value	Sig
Single measures	0.573	0.255 to 0.952	21.17	0.000
Average measures	0.953	0.837 to 0.997	21.17	0.000

Two-way mixed effects model where both people effects and measures effects are random, CI=Confidence Interval

#### Intra-rater reliablity

The difference was statistically not significant (i.e., the null hypothesis could not be rejected) for all six angles of the pROM assessment. (Table 14).

**Table 14:** Intra-rater reliability for pROM (Wilcoxon test)

Presented angle [^∘^]	Extension			Flexion		
	0^∘^	20^∘^ (a)	30^∘^ (b)	120^∘^	110^∘^ (a)	105^∘^ (b)
	p= 0.655	p= 0.200	p= 0.052	p= 1.000	p= 0.068	p= 0.244
	z= 0.447	z= -1.282	z= -1.941	z= 0.000	z= 1.824	z= 1.165

pROM results from the tasks ROM and RPM are compared (ROM-RPM)

### Task related questions

**Fig. 6:**
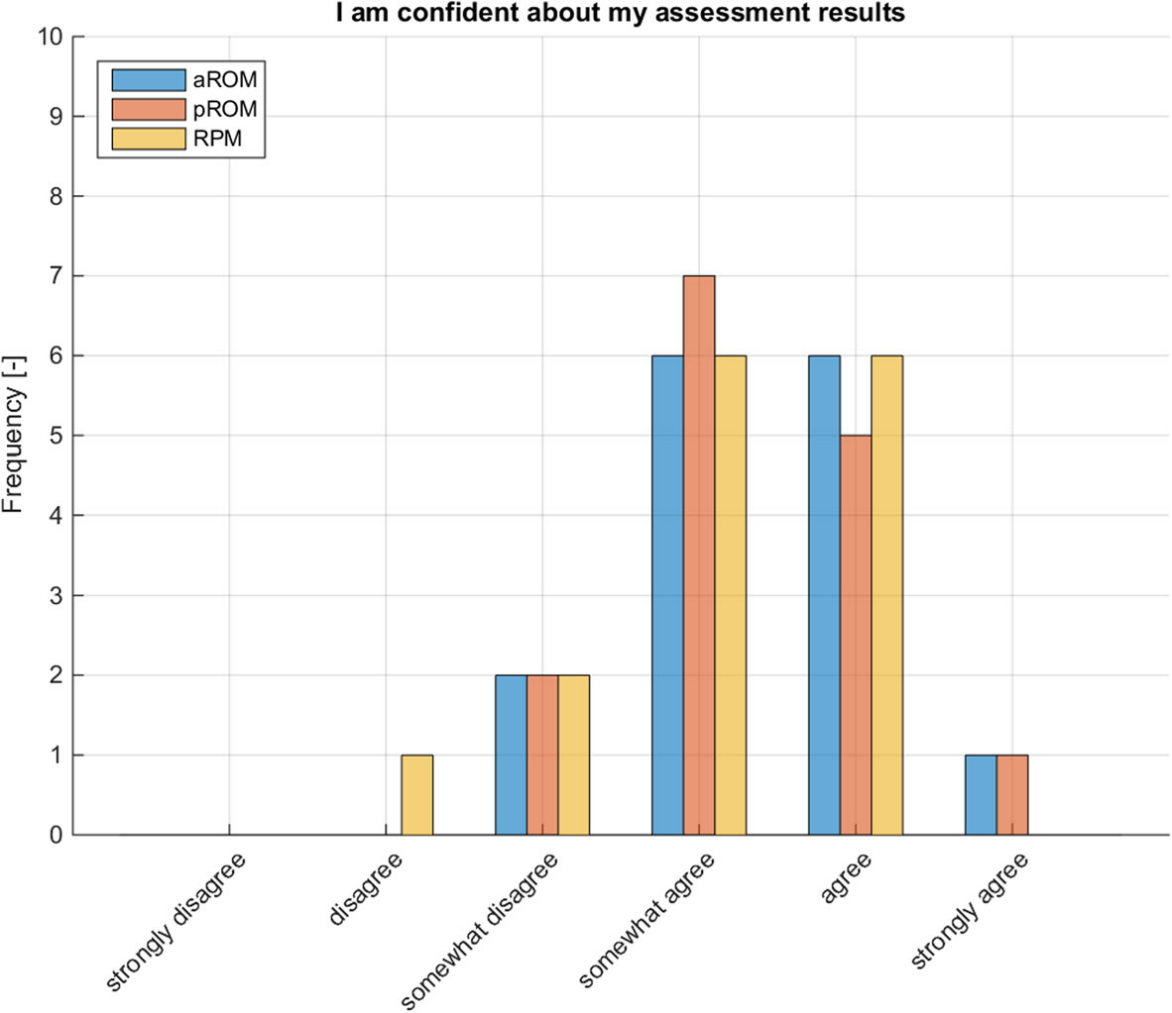
Self-assessment regarding confidence in assessment (N=15)

In aROM, pROM and RPM, the mode regarding self-assessed confidence (questions 1 to 3 in Table 15) was “somewhat agree” (see Table 15 and Fig. 6). No correlation was found between the individual’s subjective confidence in own assessment and the average error in assessment. (aROM (error quantification): R2=0.157, p=0.144; pROM (error quantification): R2=0.011, p=0.706; RPM (mTS): R2=0.001, p=0.912).

**Table 15:** Self-assessment regarding confidence in assessment

	Mean*	Mode*	SD*
1. I am confident about my pROM assessment results	3.33	3	0.82
2. I am confident about my aROM assessment results	3.40	3	0.83
3. I am confident about my RPM assessment results	3.13	3	0.92)
4. I experienced the subject‘s capabilities at my own arm in aROM	4.27	4	0.59
5. I experienced the subject‘s capabilities at my own arm in pROM	4.00	4	0.76
6. I experienced the subject‘s capabilities at my own arm in RPM	3.73	4	0.96
7. I experienced the subject‘s capabilities at my own arm in SYN	3.93	4	0.80
8. I experienced the subject‘s capabilities at my own arm in QOM	3.67	4	0.98
9. I felt myself put into the subjects’ position in aROM	3.60	4	0.91
10. I felt myself put into the subjects’ position in pROM	3.67	4	0.98
11. I felt myself put into the subjects’ position in RPM	3.47	4	1.10
12. I felt myself put into the subjects’ position in SYN	3.80	4	0.94
13. I felt myself put into the subjects’ position in QOM	3.53	3	1.10

*0 = strongly disagree; 1 = disagree; 2 = somewhat disagree; 3 = somewhat agree; 4 = agree; 5 = strongly agree

**Table 16:** End-of-study questions

	Mean*	Mode*	SD*
The warm up phase was sufficiently timed to familiarize with the device.	4.20	4	0.56
The opportunity to experience the initiation and reaction of movements by means of a robot and thus gaining an impression of the clinical condition of a patient is fundamentally enriching.	3.93	4	0.88
“Beam-Me-In” is an appropriate tool to gain an insight into the clinical picture of a patient.	3.47	4	0.83
“Beam-Me-In” opens up a new way of therapist-patient interaction.	4.13	4	0.74
The fact that I can put myself in the situation of the individual patient allows me to detect the individual patient’s problems.	3.07	3	1.03
I can imagine that the “Beam-Me-In” approach might promote the social interaction between the therapist and the patient during robot-assisted training.	3.13	4	1.25
“Beam-Me-In” can be a useful medium for teaching and learning during therapeutic education to give students an insight into the clinical picture of a patient.	3.67	5	1.40
“Beam-Me-In” opens up a new perspective. It can contribute to better empathize with the patient’s problems.	3.40	3	1.06
“Telerehabilitation” allows for a remote interaction between the therapist and the patient over a spatial distance. “Beam-Me-In” is suitable for this purpose because the therapist is enabled to assess and evaluate the progress without seeing the patient (Teleassessment: ROM, RPM, QOM, SYN).	3.60	4	0.91

*0 = not at all; 1 = not very; 2 = mildly; 3 = moderately; 4 = very; 5 = extremely

**Fig. 7:**
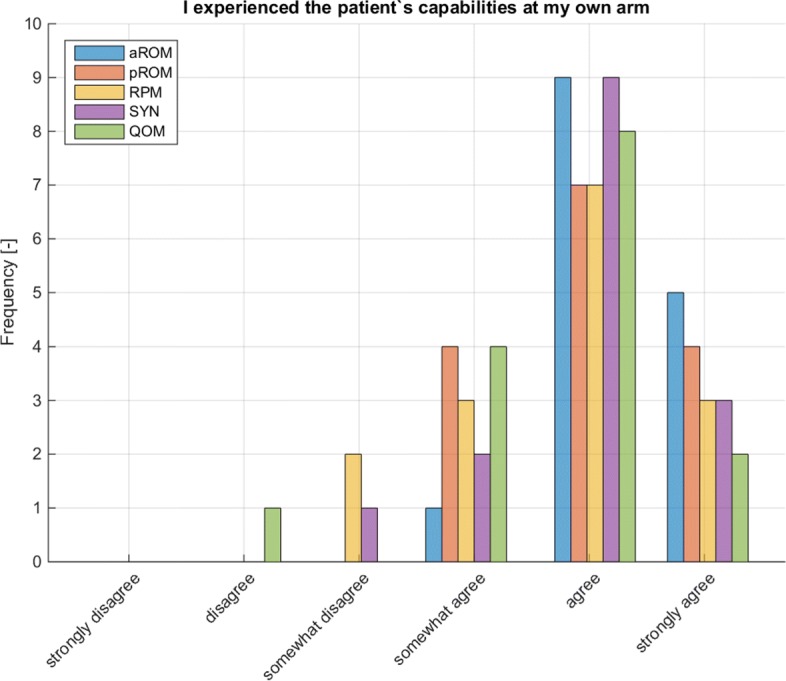
Self-assessment regarding experiencing patient’s capabilities (N=15)

In three of four tasks, the mode regarding self-assessed experience of subject’s capabilities (questions 4 to 8 in Table 15) was “agree”; the only exception was in QOM: “somewhat agree” (Table 15 and Fig. 7). No correlation was found between the individual’s experience of subject’s capabilities (Fig. 5) and the assessment results of the task (aROM (error quantification): R2=0.097, p= 0.259; pROM (error quantification): R2= 0.064, p= 0.364; RPM (mTS): R2= 0.123, p= 0.200)

**Fig. 8:**
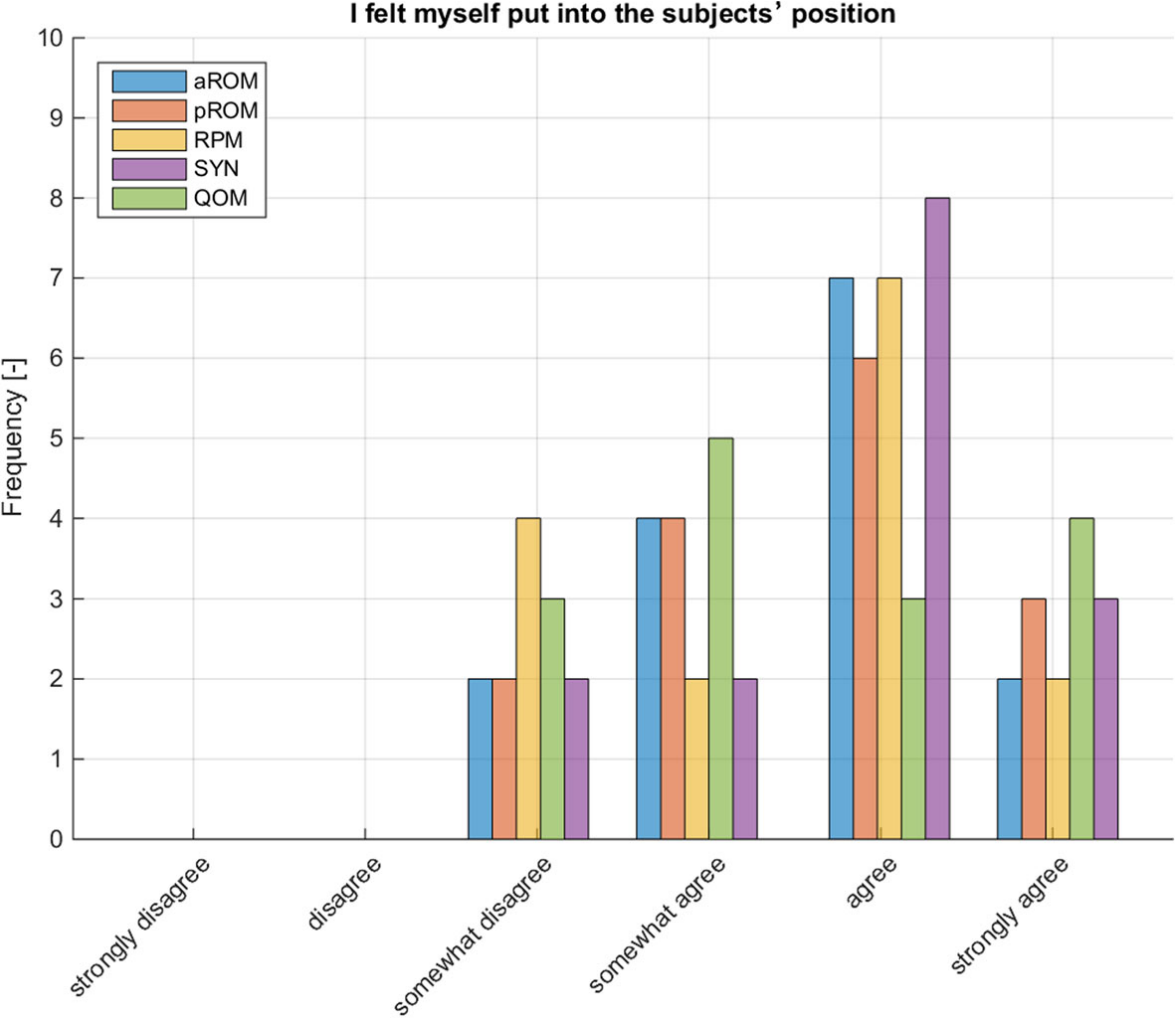
Self-assessment regarding reification during assessment (N=15)

In all four tasks, the mode regarding self-assessed reification experience (questions 9 to 13 in Table 15) was “agree” (see Table 15 and Fig. 8). No correlation was found between the individual’s reification experience and the assessment results of the task (aROM (error quantification): R2=0.082, p= 0.302; pROM (error quantification): R2= 0.038, p= 0.485; RPM (mTS): R2= 0.005, p= 0.797).

### End-of-study questions

The mode regarding self-assessed experience of subject’s capabilities within the participants was “agree”; only exceptions were “Empathy with patient’s problems” (“moderately agree”) and “‘Beam-Me-In’ as useful medium for teaching and learning” (“extremely agree) (Table 16).

## Discussion

We successfully tested the bidirectional control using two seven DOF exoskeleton robots in a teleassessment scenario with therapists. The aim was not to enable the therapist to assess the patient’s motor function remotely. Our aim was to evaluate whether a therapist could feel the patient’s disability in his arm and use clinical assessment tools, to quantify this “Beam-Me-In” strategy. We consciously limited the robotic feedback for the therapist to haptic feedback, not providing any numbers assessed by the ARMin rehabilitation robot. We showed that therapists could distinguish between different simulated movements of healthy subjects and patients post-stroke by means of the robot only, without directly touching the patient’s arm and regardless of the limited information provided by the robotic system. Thus, the “Beam-Me-In” strategy accounts for the therapist’s desire for haptic interaction as a component of hands-on therapy even with robotic technologies.

The approach to “Beam-Me-In” was consistently rated positive. However, most therapists only partly agreed on both that they could put themselves into the patients’ situation (i.e., reification) and that this allowed detecting the individual patient problems. The limited perceived reification may be explained by the mainly simulated performances in the four tasks. Nevertheless, “Beam-Me-In” was rated as a useful medium for assessment, therapy, teaching and learning during therapeutic education. It may give students insights into the clinical picture of a patient. Furthermore, “Beam-Me-In” was seen as a suitable tool during telerehabilitation. Therefore, the “Beam-Me-In” strategy has the potential to overcome reluctances towards robot-assisted rehabilitation that were presented in the introduction.

The generalizability of the positive ratings by the therapists in the end-of-study questionnaire may be limited. A demand effect through the future-oriented wording and the observed therapists’ positive attitude towards robotic devices may bias the results.

Despite the small sample size and limited generalizability, first conclusions could be drawn regarding applicability, reliability and limitations of the “Beam-Me-In” strategy for assessment. All participants were able to understand and perform the different assessment tools.

### ROM

#### Assessment evaluation

The results of ROM are satisfactory but not precise enough to be used for clinical assessment. By asking participants to quantify the end positions in each joint, we tested for the therapists’ proprioception and showed that they could quantify the limits of joint motion in the range of joint assessment using a goniometer (mean absolute error of 4.9^∘^) [34]. The participants were attached by soft cuffs to the ARMin and therefore, might have repositioned their arm slightly. Nevertheless, the ability to differentiate spans of joint motion (e.g., limits of joint motion of 5 degrees to 120 degrees results in a span of joint motion of 115 degrees) was reliable up to a span of 15^∘^. Furthermore, participants had more difficulties when assessing angles in the mid-range. During the short warm-up phase, the participants were introduced to the limits of ARMin (0^∘^, 120^∘^) and were probably orientating on these reference points, which made it easier for them to assess these values.

The excellent intra-rater reliability confirms results with standard goniometers where ROM measurements are reliable over time [35]. It is also in accordance with the task-related question (i.e., whether therapists felt confident with their ROM results) demonstrating high confidence levels.

#### Applicability

A robot can quantify ROM in a higher resolution than a therapist. A limitation of our study is that the limits of a subject’s movement in pROM were simulated by a simple spring-damper element at the patient limits, which did not consider biomechanical limitations, e.g., stretching of soft tissues and the resting tone of the muscles. Furthermore, the therapist could not influence the subject’s movement pattern during aROM assessment. By controlling the movement pattern (e.g. take more time to explore the limits) a therapist could have had more time to identify the angle.

### RPM

#### Assessment evaluation

The participants achieved excellent reliability scores in the assessment of mTS. Participants were able to feel the reaction to an imposed movement without directly placing hands on the subject. The quantification of the catch angle showed similar fair reliability as the pROM angles in the mid-range with errors up to 15^∘^. The catch could be identified by most (14 of 15) of the participants.

#### Applicability

The speed of movement is critical when assessing RPM as both the joint angle and muscle reaction are velocity dependent. An increase in stretch velocity results in an increase in resistance to passive movement that we considered and implemented in our strategy [24]. Similar to a pROM assessment, guidance of the arm by the therapist and identification of a limitation in movement by the therapist is required for that assessment. Therefore, an automated interpretation by the robot is rather difficult, it demands therapist experience to react on the patients arm behavior. The “Beam-Me-In” strategy complements the clinical assessment with the possibility to assess RPM remotely.

With robotic training RPM decreases for a certain time window, as do pain and perception of arm heaviness [36]. Therefore, identification of RPM during the movement training itself can provide additional information to the therapist. The therapist can then adapt the training accordingly and choose training tasks that are suitable for a specific hypertonic status.

### SYN

#### Assessment evaluation

Assessing the abilitiy to fractionate a movement synergy, the participants achieved excellent reliability scores and were able to differentiate three patient-like movement patterns from each other. This result has to be put into perspective since the subjects were simulated with no patient induced noise overlain, i.e., non-smooth movement patterns of higher frequency.

#### Applicability

Compared to end-effector based devices, exoskeleton rehabilitation devices provide measurements of single joints of a patient’s arm. Therefore, “Beam-Me-In” provides an excellent tool to measure and present arm synergies and further abnormal movement patterns.

### QOM

#### Assessment evaluation

The participants were able to distinguish between small differences in smoothness and accuracy. For smoothness in particular, the participants’ quantification seems to correlate well to the smoothness calculated according to Balasubramanian et al. [31]. However, for both, smoothness and accuracy, the results were limited regarding inter-rater reliability.

#### Applicability

The two parameters smoothness and accuracy are hardly ever quantified in clinical routine. Unexpectedly, therapists were in average able to score smoothness and accuracy differentiating between slightly different movement patterns. Therefore, different movement patterns of different smoothness and accuracy can be haptically displayed by ARMin and interpreted by a therapist remotely using the “Beam-Me-In” strategy. However, to increase the inter-rater reliability the backlash between human arm and the cuffs needs to be reduced. A therapist could not clearly say if the “non-smooth” or “non-accurate” movement is due to the subject’s performance or due to the participant’s own freedom to move within the robot. For optimal application of the “Beam-Me-In” strategy, future redesigns of the ARMin robot should consider an undisturbed transfer of the movements between robot and human arm.

### General remarks

To assure consistency of the conditions among the therapists, subjects in most task were only simulated. The simulated impairments were not validated or compared to recorded impairments. Nevertheless, the therapists were able to quantify the simulated biofeedback in all four types of assessments. In a next step, patients should be integrated into the task to allow for real patient-therapist interaction and to obtain opinion of patients about this new form of telerehabilitation. While the simulations in this work do not necessarily reflect actual impairments with a quantifiable clinical relevance, this study shows that simulated impairments may be a feasible method to determine the efficacy of haptic feedback. The feasibility is supported by the therapists’ ability to quantify the simulated biofeedback in all four types of assessment (i.e., reduced active and passive ROM, resistance to passive movement, lack of ability to fractionate a movement, and disturbed quality of movement). Furthermore, the resolution of the abnormal movement patterns should be increased since the here presented results only prove that by using the “Beam-Me-In” strategy therapists are able to distinguish between extreme cases. The “Beam-Me-In” strategy is not limited to the presented assessments. Although we did not test for muscle strength, it could be easily implemented as an assessment. From educational point of view, further movement abnormalities, such as “clonus”, could complement the RPM and SYN experience, as suggested by the therapists.

The “Beam-Me-In” strategy provides a unique application of telerehabilitation where an exchange of haptic information over distance in real-time is enabled through two exoskeletons. As we connect both the patient and the therapist to a device, we create a human-robot-robot-human interaction. In this study, the application was concentrated on assessment but could easily be extended to task oriented training. Furthermore, the “Beam-Me-In” strategy could be applied to other diseases, such as multiple sclerosis or spinal cord injury, and for other types of training, such as task oriented training using remote robotic devices.

The here presented “Beam-Me-In” strategy is not restricted to the ARMin device and can be transferred to contemporary available robotic solutions. However, exoskeleton robots with high DOF are expensive devices compared to end-effector devices or exoskeleton robots with a low number of DOF. Since both, a high number of features and low costs, are desired by therapy providers, the cost effectiveness of the suggested setting has to be discussed [16]. The combination of robotic solutions of lower costs with a more specific functionality for the master or the slave role and with only one to three DOF might be suggested for current clinical use. We expect that production costs for exoskeleton robots will decrease in the future and the suggested multi-DOF exoskeleton setting might then be considered.

## Conclusion

The “Beam-Me-In” strategy allows for remote haptic interaction between the therapist and the patient. We could show that information about joint position, resistance to passive movement, inter-joint coordination, smoothness and accuracy during a point-to-point reaching task can be transferred to the therapist’s own arm and allows him or her to assess these parameters. In particular, for the identification of abnormal movement patterns that need to be induced by passively moving the patient, “Beam-Me-In” offers a tool for remote assessment that is superior to the robot alone. For feasibility testing, we limited the resolution to provide patient impairments representing the entire patient population. As a next step, we would test the “Beam-Me-In” strategy with higher resolution of abnormal movement patterns and also test the strategy with therapists and real patients in a clinical setting.

We conclude that the “Beam-Me-In” strategy is a new opportunity to assess and train patients. The “Beam-Me-In” strategy offers a possibility to experience a new way of therapist-patient interaction. Therapists can subjectively assess movement characteristics of a subject via realistic haptic feedback through a seven degree-of-freedom exoskeleton. Our system does not replace the robot-based quantification of the health status which is sensitive to smallest changes. It rather aims to complement the information provided to the therapist. In combination with automated robot-assisted assessment the “Beam-Me-In” strategy may offer a complete tool to assess stroke patients remotely. The “Beam-Me-In” strategy device has the potential to provide valuable and sophisticated haptic feedback that will help address the barriers to implementing robot-assisted telerehabilitation.

## Data Availability

Please contact corresponding author.
